# Supplementary material for: Clinical Markers of Chronic Hypoxemia in Respiratory Patients Residing at Moderate Altitude
Source: Life (Basel). 2021 May 10;11(5):428. doi: 10.3390/life11050428 (PMC8150591; doi:10.3390/life11050428)

life

MDPI

Clinical Markers of Chronic Hypoxemia in Respiratory Patients Residing at Moderate Altitude

Rosario Fernández-Plata [1], Ireri Thirion-Romero [2], Karol J. Nava-Quiroz [3], Gloria Pérez-Rubio [3], Sebastián Rodríguez-Llamazares [2], Midori Pérez-Kawabe [2], Yadira Rodríguez-Reyes [4], Selene Guerrero-Zuñiga [4], Arturo Orea-Tejeda [5], Ramcés Falfán-Valencia [3,*] and Rogelio Pérez-Padilla [2,*] on behalf of the Mexican Translational Research Hypoxemia Working Group [†]

Table S1. Informative Ancestry Markers (AIMs) and reference populations included.

		ZAP n = 60			CEU n = 120			D	HYP n = 92		
**Chr**	**SNP**	**A1**	**A2**	**MAF**	**A1**	**A2**	**MAF**		**A1**	**A2**	**MAF**
1	**rs4528122**	T	C	0.067	C	T	0.142	0.792	C	T	0.386
1	**rs986690**	G	A	0.017	A	G	0.25	0.733	G	A	0.28
4	**rs10516422**	G	A	0.283	G	A	0.017	0.267	G	A	0.246
5	**rs10515716**	T	C	0.267	C	T	0.208	0.525	C	T	0.386
6	**rs1878071**	A	C	0.317	C	A	0.217	0.467	C	A	0.444
9	**rs4084051**	T	C	0.25	C	T	0.175	0.575	C	T	0.488
9	**rs7853112**	C	A	0.25	A	C	0.35	0.4	A	C	0.404
9	**rs10511491**	C	T	0.25	T	C	0.391	0.358	C	T	0.401
9	**rs1039336**	A	G	0.133	G	A	0.242	0.625	A	G	0.305
9	**rs10116714**	A	G	0.183	G	A	0.05	0.767	G	A	0.440
9	**rs1980888**	G	A	0.033	A	G	0.1	0.866	A	G	0.325
9	**rs4743556**	C	T	0.172	T	C	0.167	0.661	C	T	0.456
12	**rs6487927**	C	T	0.033	C	T	0.475	-0.442	T	C	0.256
13	**rs2147155**	0	T	0	G	T	0.5	-0.5	G	T	0.119

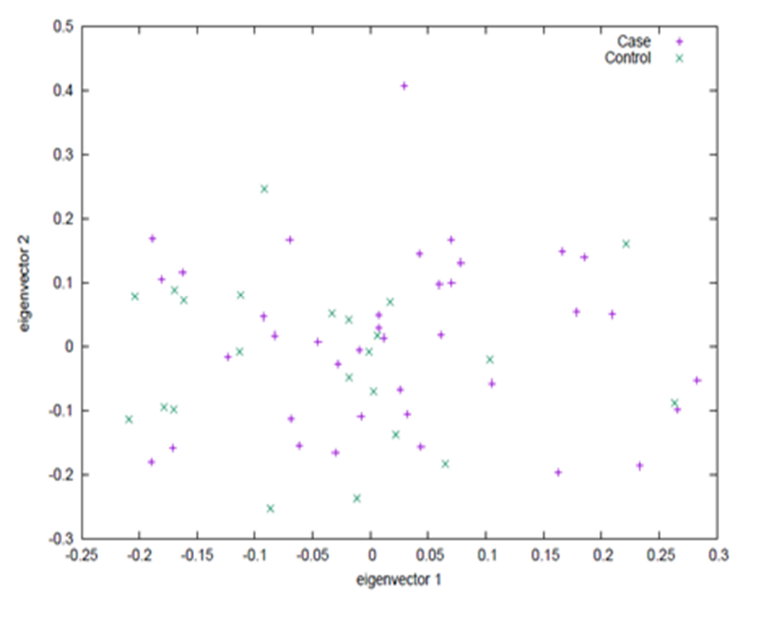

Supplement: Supplementary file 1 [file life-11-00428-s001.zip › life-1179536-supplementary.pdf]
